# A deep neural network approach for sentiment analysis of medically related texts: an analysis of tweets related to concussions in sports

**DOI:** 10.1186/s40708-021-00134-4

**Published:** 2021-07-01

**Authors:** Kayvan Tirdad, Alex Dela Cruz, Alireza Sadeghian, Michael Cusimano

**Affiliations:** 1 Department of Computer Science, Ryerson University, Toronto, Canada; 2 Li Ka Shing Knowledge Institute, St. Michael’s Hospital, Toronto, Canada; 3 Department of Surgery, University of Toronto, Toronto, Canada

**Keywords:** Artificial intelligence, Machine learning, Deep learning, Sentiment analysis, Traumatic brain injuries, Concussion

## Abstract

Annually, over three million people in North America suffer concussions. Every age group is susceptible to concussion, but youth involved in sporting activities are particularly vulnerable, with about 6% of all youth suffering a concussion annually. Youth who suffer concussion have also been shown to have higher rates of suicidal ideation, substance and alcohol use, and violent behaviors. A significant body of research over the last decade has led to changes in policies and laws intended to reduce the incidence and burden of concussions. However, it is also clear that youth engaging in high-risk activities like sport often underreport concussion, while others may embellish reports for specific purposes. For such policies and laws to work, they must operate effectively within a facilitative social context so understanding the culture around concussion becomes essential to reducing concussion and its consequences. We present an automated deep neural network approach to analyze tweets with sport-related concussion context to identify the general public’s sentiment towards concerns in sport-related concussion. A single-layer and multi-layer convolutional neural networks, Long Short-Term Memory (LSTM) networks, and Bidirectional LSTM were trained to classify the sentiments of the tweets. Afterwards, we train an ensemble model to aggregate the predictions of our networks to provide a final decision of the tweet’s sentiment. The system achieves an evaluation F1 score of 62.71% based on Precision and Recall. The trained system is then used to analyze the tweets in the FIFA World Cup 2018 to measure audience reaction to events involving concussion. The neural network system provides an understanding of the culture around concussion through sentiment analysis.

## Introduction

Traumatic brain injuries claim 60,000 lives, and three million people sustain concussions and other forms of mild traumatic brain injuries (mTBI) annually in North America [1–3]. Sport-related head trauma is the most common mechanism of mTBI in youth, and more than a half million North American youth under the age of 15 require hospital-based care annually [3], and these doubled during the period from 2001 to 2012 [4]. The Centre for Disease Control (CDC) declared that concussions are reaching “epidemic levels” and deserve further research [1]. Concussion, a form of mTBI, can have adverse impact upon cognitive, emotional and social functioning, with lasting personal, familial, and societal implications. For adolescents with concussion, mental health issues compound matters due to the significant cognitive development and psychosocial and emotional growth that occurs concurrently [5]. The potential cumulative or long-term deleterious effects of concussions are worrisome [6–11], as repeated concussions may be related to degenerative neurological conditions later in life [12–14]. Of equal concern, the adolescent years are often marked by engagement in alcohol and drug use, both of which may influence the incidence and outcomes of all forms of TBI. While concussion may be an important antecedent to prolonged cognitive, emotional, and behavioral difficulties, most patients rarely show overt manifestations beyond a week [15], and there still exists (particularly amongst the sports community) a widespread attitude that concussions are “a part of the game” and a sign of “toughness” [16]. There is often a premium put on a speedy return to play for athletes, which can hinder prevention and treatment efforts, as well as a pressure to return too soon to a pre-concussion learning regimen [17].

It is also understood that many instances of concussions have remained unreported [18–20]. Research shows that athletes of different sexes and those in competitive versus recreational sport view concussion and activities like “return to play” quite differently. For example, in interviews of youth ice hockey players, we found that although hockey players from leagues that allowed body checking often underreported injuries, female players and boys from non-body checking leagues almost never underreported their injuries [18]. Aspects of hockey culture such as an overemphasis on winning games and upheld misperceptions about the risks associated with concussion were identified as relevant to the underreporting of concussions. This is of concern because individuals who sustain multiple concussions before recovering from the first concussion may be at risk of a potentially fatal or disabling “second impact syndrome”. Various factors relevant to the underreporting of concussions include player’s motivation to win, group membership dynamics such as a player’s role as the team’s “enforcer,” coaches’ own motivation to win to further their own opportunities in the sport, and parents’ personal financial interest or alternative agenda in terms of time commitments and their child’s future career prospects. This and other work indicates that underreporting is prevalent and associated with misconceptions about injury risk and a culture that both reinforces and encourages underreporting with tacit or overt complicity of parents and coaches. This would suggest the need to alter the culture of violence and tough play in sport by education, rule changes, economic measures, and changes in governance of the sport.

In response to these concerns, a number of agencies have developed protocols to minimize the burden and risk of concussions [17]. In fact, laws have been instituted in a number of jurisdictions to reduce the burden of concussions [21, 22]. These laws mandate the removal from play of any youth athlete suspected to have a concussion and makes concussion education mandatory for certain individuals involved with youth sport. However, larger issues within sport culture may limit the effectiveness of such laws and potentially render them ineffective. So understanding and changing the culture around sports-related concussion can be particularly important to the effectiveness of any intervention intended to reduce concussion, such as new legislation [23].

Measuring the culture around society’s understanding of concussion and the sentiment towards concussion is the focus of this work. Traditional means to collect and understand the general public’s opinion on sports-related concussions include surveys, interviews, and questionnaires. These methods are time-consuming and may also introduce unintended bias based on the specific questions asked and the inclusion list used to select potential survey candidates [24, 25]. These methods can also become unfeasible if wide segments of the population are sampled in order to get a representative sample of opinions of people. One of the objectives of this work is, hence, to develop a feasible system that can understand and measure the public’s current opinion on sports-related concussion by analyzing an extensive dataset of the public’s viewpoints and sentiments around concussion. The information provided by this system can then be used by a variety of stakeholders to gauge public opinion and attitudes towards concussion. This information could be useful in the successful implementation of strategies to reduce the burdens of concussion.

In this work, we harness the power of modern social media to provide valuable insight into the culture surrounding sports-related concussion. Twitter is a popular social media platform that averages 330 million active users that post opinions on various topics [26]. Analysis and mining of Twitter data can provide insight into the beliefs and attitudes towards health-related concerns, which may directly or indirectly affect patients’ decisions [27–29]. While twitter provides a large pool of data, the volume of data generated from the platform can be very problematic for the traditional analysis approach. For example, a current trending topic can generate a volume of tweets from various users in a short time that manual analysis of tweets can quickly become unfeasible.

In our previous related work, we explored Twitter as an effective platform to understand public perception of sport-related TBI [30]. A content and sentiment analysis of 7,483 tweets was performed manually, and we demonstrated a mismatch between the scientific community’s perception of concussion and that of the public. Other works have demonstrated the applicability of deep learning methods to mining biological data such as images, signals, and sequences [31]. Therefore, the present work further enhances our previous approach and develops an automated deep learning-based system to analyze the tweets of sports-related concussions to determine the general public’s sentiment towards concussion in sports.

Our goal was to develop a system to perform automatic annotation of sports-related concussion tweets as either positive (illustrates an understanding of the serious health risk of TBI), negative (disregard for, lack of concern for, or lack of understanding of TBI), or neutral (impartial attitude toward TBI or no clear effect regarding TBI in sports). We utilize a word embedding approach that contains an ensemble of semantical, syntactical and sentimental representation of each word. An ensemble neural network is used to consolidate the outputs of seven deep neural network models to classify the sentiment of a tweet. This work aims to aid further research in the area of concussions in sports by providing a system to automate the sentiment analysis of extensive data. In this work, we assess our developed system by also analyzing the 2018 FIFA world cup by utilizing the automated system to determine the current sentiment of concussions relating to football.

The remainder of this paper is organized as follows. Section 2 provides a description of earlier similar work. Section 3 provides an explanation of Twitter data and the key components and the labeling related to this work. Section 4 explains the proposed deep learning architecture. Section 5 describes our implementation. Section 6 provides details of the evolution and results, and Section 7 provides a summary of the work.

## Related works

Earlier related prior research has been conducted in the area of concussion and automated sentiment analysis. However, they have primarily focused on one or the other.

Some works [32, 33] have examined traumatic brain injuries, but do not explore the impact and correlation of sports-related activity to the injuries. The work in [34] reports an analysis of unreported concussion for high school football players. This work illustrates a substantial rate of concussions being unreported by surveying 20 high school football teams. It also provides an insight into the culture of sports and how these may influence players to keep concussions hidden. The work, however, only examines a small set of the population by limiting its survey to 20 schools. Also, it is observed that the process of scaling the methodology of the research to a broader national or global scope will be very costly. Another work [35] presents an analysis of concussion events during the 2014 FIFA world cup, where the video footage of the 64 games have been analyzed, and 67 instances of athletics exhibiting signs of concussions not being considered for medical assessments are identified. The same research group reports the first work in analyzing concussion via Twitter data [30]. They examined concussion-related sports tweets manually from June to July 2013 and categorized each tweet into three sentiments (positive, negative, and neutral). However, the manual method of analyzing tweets can be very time-consuming, and as a result, the amount of data that processed was not enough for further analysis.

Many sentiment analyses on Twitter data have been conducted but have been limited to traditional natural language processing and machine learning algorithms [36–39]. Automated sentiment analysis systems on Twitter data were presented by several groups [40–42]. All presented work utilized the neural network systems to produce state-of-the-art performance. However, all three works focused on the sentiment of general topics rather than specifically concussion-related sports injuries. This presents a problem as the neural network sentiment analysis is commonly domain-specific. Another work [43] also examined sentiment analysis on Twitter data of the World Cup soccer 2014, but explore the overall sentiment of the event and not concussion specific.

In another work, an ensemble system consisting of two similar convolutional neural networks (CNN), differentiated only by the initialization of their word embedding, was presented. Both networks include two layers of convolution and a max-pooling layer followed by a fully connected layer and SoftMax output layer. However, the convolution and max-pooling parameters used in the Word2Vec embedding differ between models [40]. Similarly, another ensemble system that consists of 10 CNN and 10 bidirectional long short-term memory (LSTM) networks together with both Word2Vec [44] and FastText models [45, 46] for word embedding was reported in [41].

Another project illustrates a two-layer bidirectional LSTM with attention gates where a GloVe model [47] to embed their words was pre-trained. This system treats the tweet as a sequence of text with LSTM while also learning which region of the tweet to emphasize via the attention gate [42].

Sullivan et al. provide an analysis of concussion-related tweets by mining and analyzing them in a span of 7 days [48]. This work illustrates the capacity of Twitter data as a medium for sports concussion information and education [48]. However, the proposed method of manual annotation of data is not viable in capturing the broader population’s beliefs and attitudes. Different from our work, they propose a custom coding scheme to manually analyze the content of the tweet to determine the major themes rather than an automated procedure to determine the sentiment of the tweet.

Prior works in sentiment analysis have primarily utilized neural network models that contained a single vector embedding. Such that the embedding model within their preprocessing stage only captured a single representation of distance based on co-occurrence of words. Therefore, other linguistic features such as part-of-speech or the semantic orientation are never encoded in the vector representation of a word. Thus, the neural network models never see the additional linguistic features.

## Twitter data, their components and tagging (labeling)

Twitter users share social media messages with other users through Tweets. Tweets are short informal messages that can contain a combination of media components such as hashtags, mentions, emoticons, and URLs. We describe the different components and provide examples for each in Table 1. Tweets allow the users to freely create a message using text and any combination of media components, so long as the combined components and text do not exceed 280 characters. The Twitter user who posts a tweet is called the author, and they have the option to post a general tweet, reply, or retweet. A general tweet is an original message that the author post, while a retweet is a resharing of a post from another user. Reply tweets are similar to a general tweet, but the content of the message pertains to a reply to another tweet, while the topic of a general tweet does not directly relate to another tweet. Retweets are prepended with an ‘RT’ token, providing a clear distinction between retweets and general tweets.

Similar to [30], we categorize each tweet into 3 different classes (positive, negative, or neutral) based on the author’s sentiment pertaining to the tweet. Sentiment is based on the level of recognition or disregard to the risk of TBI that the author demonstrates within the tweet. Positive sentiment indicates that the author demonstrates a strong recognition of the risk associated with TBI. Negative sentiment illustrates that the author demonstrates a disregard of the risk associated with TBI, while neutral provides no sentimental opinion. Table 2 provides a detailed description of each class and some example tweets.

**Table 1:** List of acceptable components in a tweet. For each example, we bold the described component within the tweet

Component	Description	Example Tweet
Hashtag	Metadata tags are used to allow a user to apply dynamic user-generated keyword tags. Hashtag are introduced by the hash symbol “#”. It is then followed with the hashtag context which comprises of one or more keywords.	Damn!!! Out cold!! Awesome knockout @KingMoFH**#BellatorMMA** RT @ConcussHealth: RT @keithmxatc: Offseason **#concussion** woes of NFL players a scary reminder of a serious problem | Shutdown Corner’
Mentions	Mentions are citations to a specific twitter user via their username. It allows authors to mention a specific user in their tweet, providing more visibility of the tweet to the mentioned user. Mentions are identified by their preceding “@” prior to the username.	**@The_Meek** while playing “the hockeys” last night I took a puck to the head. It was epic. I think I’m concussed. **@TSNBobMcKenzie** why not? Hit to the head, right. #shannysajoke
URLs	Emoticons are emotion icons that provide a pictorial representation of a facial expression and allow users to express a feeling or mood. They are formed using punctuation marks, numbers and letters.	got a slap shot to the head with no helmet, got the biggest headache :’( RT @Mike_Davis125: Won my fight by RNC he went out cold! Yay and still champion 8 wins in a row :) #mma #ufc #champion http://t.co/KdMGOZ5wÆ’
Emoticons	URLs are links They are commonly linked to a photo, Gif, or video relating to the content of the tweet.	Steelers erred on the side of caution with concussion in 2009: The defending Super Bowl champions were against... **http://t.co/DlT4aSB0tV** No suspension for this? Are you kidding me? Its a hit to the head! **http://t.co/ffpFGD23MX**

**Table 2:** Description of Sentiment labels of Concussion Dataset

Sentiment	Description	Example
Positive	The author illustrates the severity of concussion and its superseding importance above all other sport’s related topic	RT@[user]: Playing on with a concussion isn’t big or brave, it’s sheer stupidity. (#lions) @[user] I know. Heard that he has a mild concussion. MLB needs to find a solution before something terrible happens.
Negative	The author illustrates a degrade, lack of concern or understanding of the dangers of concussion and/or places more positive sentiments towards other sport’s related topic (i.e,. result of the game)	I’m so glad Silva got his bell rung I really enjoyed seeing Anderson Silva get his clock cleaned
Neutral	The author provides no opinion about any given topic or does not illustrate a stronger positive opinion of one topic to another.	Toews is definitely concussed Cobb leaving hospital, placed on concussion list

## Methodology

**Fig. 1:**
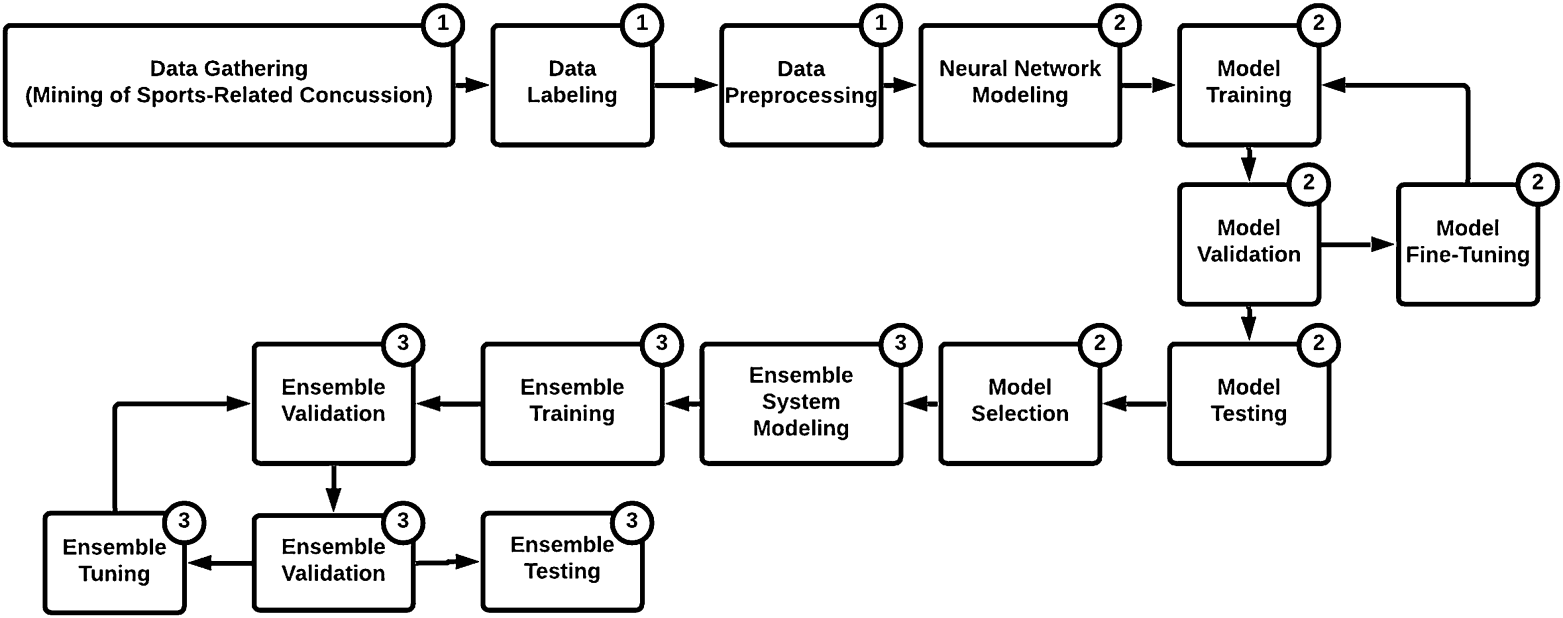
Block diagram illustrating the 3 main phases of work: (1) Data framework, (2) Deep Neural Network framework, and (3) Ensemble framework

Three main phases of work were conducted in this work that focused on data, neural network, and the ensemble system, as illustrated in Fig. 1. Our proposed architecture comprises of (i) preprocessing, (ii) deep neural networks, and (iii) an ensemble model. The first layer includes a data preprocessing unit, which contains different blocks, including cleaning, normalization and vectorization. The second component is a collection of seven deep learning neural network models, which are used to process the data and prepare the output. The third component is an ensemble model whereby the outputs from the previous layer are prioritized, and a final decision is made. In the following, we explain each layer and its respected processing blocks.

### Preprocessing

**Fig. 2:**
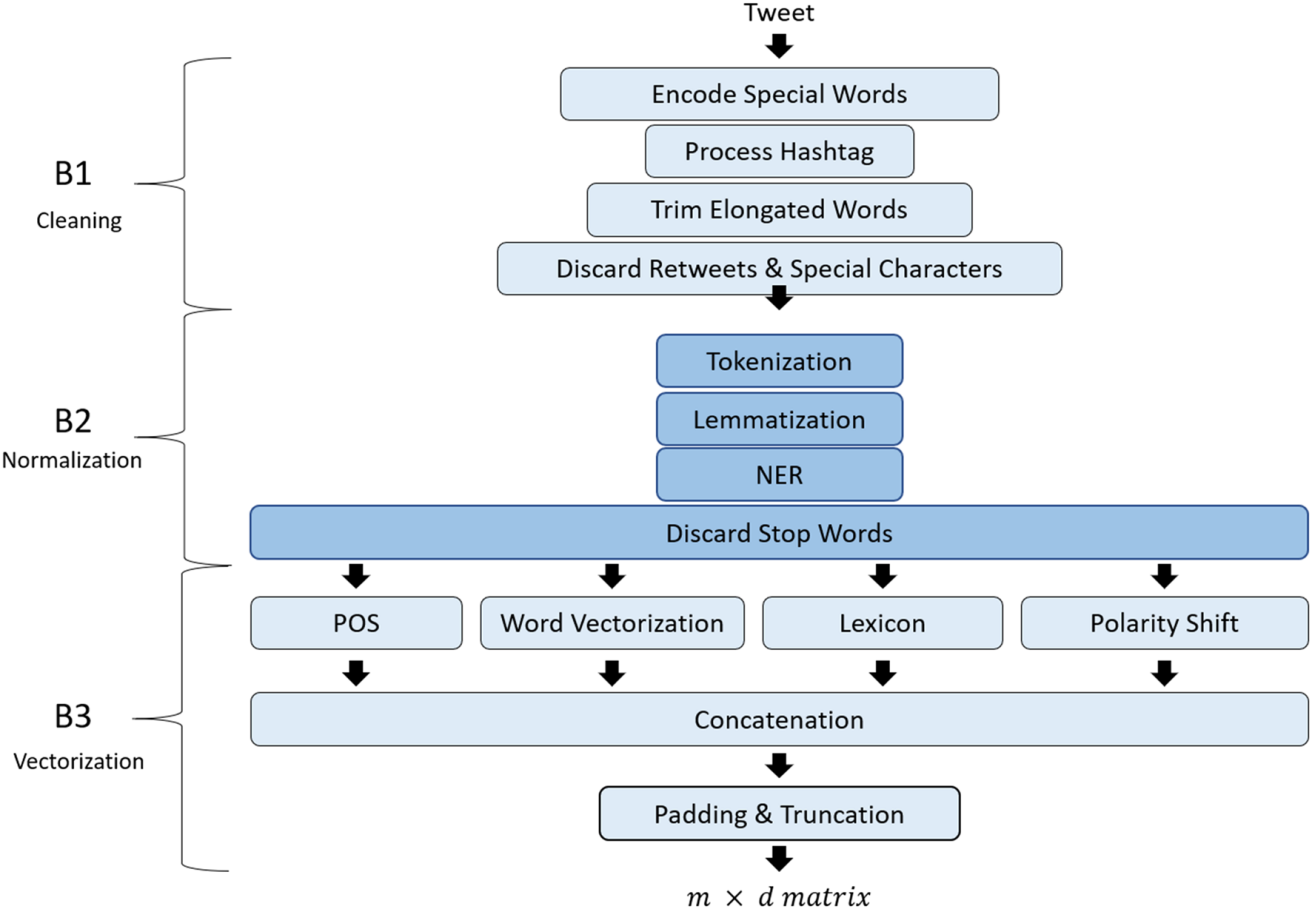
Diagram illustrating the preprocessing pipeline blocks

The preprocessing pipeline consists of three main blocks, as illustrated in Fig. 2. The first block performs general natural language preprocessing that aims to mask and clean the tweets. The second block performs a sequence of methods to normalize the tweets. Lastly, the third block in the pipeline performs vectorization of the twitter data to convert the terms into vector embedding representation.

#### Block 1—Cleaning

Since tweets are short informal text that contains slight differences due to social media elements such as emoticons, the first block cleans the tweet by removing noise introduced by the subtle changes brought on by these features. Because the variance between specific special words (i.e., emoticons) does not contribute to the semantic orientation to the tweets, special words are first encoded. For example, various smiley faces (i.e., :-|) and < 3) are all encoded to ’< smile>’. In addition to emoticons, user mentioned elements (indicated by ’@user_name’) are also encoded to a single ’< user>’ tag to remove additional noise. Hashtags are user-defined tags to provide emphasis on the keywords of the tweet. As hashtags provide a rich indication of the tweet’s main focus, we provide additional special processing of these tags. First, hashtags are identified by the hashtag symbol (#) and encoded with the unique tag ’< hashtag>’. Second, the hashtag context which follows the hashtag symbol is segmented into individual words resulting in an output of the encoded hashtag followed by one or more keywords. Additionally, since tweets are highly informal, words are sometimes elongated (i.e., helloooo), which provides additional noise complexity towards sentiment analysis. We simplify this complexity by trimming elongated words to their standard length. Instead of posting original content, twitter users can re-post tweets by other users. These tweets are marked with a retweet tag (RT) at the start of the tweet. While this tag aids in identifying if the tweet is original or not, it does not modify the sentiment of the tweet. Therefore, retweet tags are discarded along with special characters to reduce the noise complexity.

#### Block 2—Normalization

The second block normalizes each tweet to remove the sparsity introduced from various word forms due to grammatical conventions. This allows us to reduce the vocabulary complexity of our proposed system. First, tokenization is used to separate and extract each word from the tweet. After each word is extracted, lemmatization is used to convert each word into its base form. Lemmitization is the process in linguistics of grouping inflected forms of a word (e.g., “builds”, “built”, “building”) together as a single base form called the “lemma” (e.g., “build”). It is frequently used in dictionaries [49]. Besides, Lemmatization is used over stemming with the aim of ensuring additional noise is not introduced in the process. Therefore, words (e.g., ’saw’) will not merely be cropped, which could lead to inaccurate base form, but rather it is converted to their lemma (’see’ or ’saw’ based on Part Of Speech) [50]. Afterwards, Name-entity recognition (NER) [51] is applied to the word, to encode the name of things to their associated classes. For instance, the name Bob and John would both be encoded to the same tag ’<person>’. The following 7 entities are identified and classified in this current body of work: name, location, organization, money, percent, data, and time [52]. As sports-related tweets contain many different mentions of player names, teams, cities, player stats, date and time of games, NER is necessary to reduce the vocabulary complexity of the system. Lastly, we discard all stop words (i.e., the, a, and, is, etc.) to reduce the vocabulary further. Since they are highly frequent words that appear in all tweets and provide little to no contribution in discerning the sentiment of a tweet, they are discarded instead of processed into a normalized form.

#### Block 3—Vectorization

Computers do not naturally understand words but applying a real number vector representation to words, something called “word embedding”, means we can apply mathematical rules and perform matrix operations on them [53]. For example, one might create a vector that has as many dimensions as the text one is analyzing has unique words. Each unique word would be assigned a unique dimension and be represented by a 1 in that dimension with 0s everywhere else. This might mean that a word like “head” is represented by the vector [1, 0, 0,...0] and “brain” by the vector [0, 1, 0, 0,....0]. These vectors can then represent the whole body of the text and one can apply deep learning algorithms to analyze the text [53].

Prior works in sentiment analysis have primarily utilized neural network models that contained a single vector embedding such that the embedding model within their preprocessing stage only captured a single representation of distance based on co-occurrence of words. Therefore, other linguistic features such as part-of-speech, or the sentimental orientation are not encoded in the vector representation of a word. Therefore, we use a word embedding that explicitly contains syntactical, semantical, and sentimental features of the word. The ensemble word embedding is a concatenated vector containing four signal sources: part-of-speech (POS), sentimental grade, polarity shift, and semantical representation. The part-of-speech analysis is conducted on the whole tweet to classify each word into their associated Penn Treebank POS tag (36 tags) and assigned a numerical value from 1 to 36. SentiWordNet 3.0 is used to calculate the sentimental grade of positivity, negativity and neutrality of each word [54]. A scalar grade from 0 to 1 is assigned to each sentiment with the cumulative score of all three equating to one, as illustrated in the following equation:

1 $$\begin{aligned} \begin{aligned} 1=S_++S_-+\ S_n, \end{aligned} \end{aligned}$$

where $S_+$, $S_-$, and $S_n$ are the scalar value for positivity, negativity, and neutrality, respectively. A rule-based algorithm is implemented to identify the polarity shift of the given word as either normal or inverted [55]. For example, the word “happy” in the context of “I am not happy” will have an inverted polarity shift, while “happy” will have a normal polarity shift in the context of “I am happy”. A language representation algorithm is trained to learn the semantical vector representation of each word. Word2Vec algorithm was used to generate the semantical word embedding [44]. All four signal sources are then concatenated to generate a 205-dimensional(D) vector where 1, 200, 3, and 1 represent the POS, word vector, lexicon, and polarity shift, respectively. The last block in the preprocessing pipeline embeds each word using various methods (POS, word-vectorization, lexicon analysis, and polarity shift). It concatenates each embedding to produce a single vector representation of each word. Each vectorized word is then joined together again by concatenating each 205-D vector producing an *n* by 205 representation of the tweet, where *n* is the number of words in the tweet after Block 2. The value of *n* was calculated as the number of words in the longest tweet to ensure no loss in information to large tweets. Padding was applied to shorter tweets to generate a uniform input for the system.

### Neural network models

Seven different neural network architectures with various hyper-parameters have been evaluated in this work. They include FeedForward Neural Network (FFNN), Single-Layer CNN, Multi-Layer CNN, Gated Recurrent Unit (GRU), LSTM, Bidirectional LSTM, and Temporal Convolutional Network (TCN). FFNN provides fully connected layers between each layer, providing an overall general mapping between connecting layers. The CNN models are selected to capture the spatial relationship between text, while RNN models provide models to capture the temporal relationship of words within the tweet.

*FFNN:* are fully connected layers containing a layer of inputs, one or more layers of hidden neurons (computational units) and a last layer of outputs. The neurons of the prior layer are fully connected to the subsequent layer by weighted parameters that the network learns [56, 57].

**Fig. 3:**
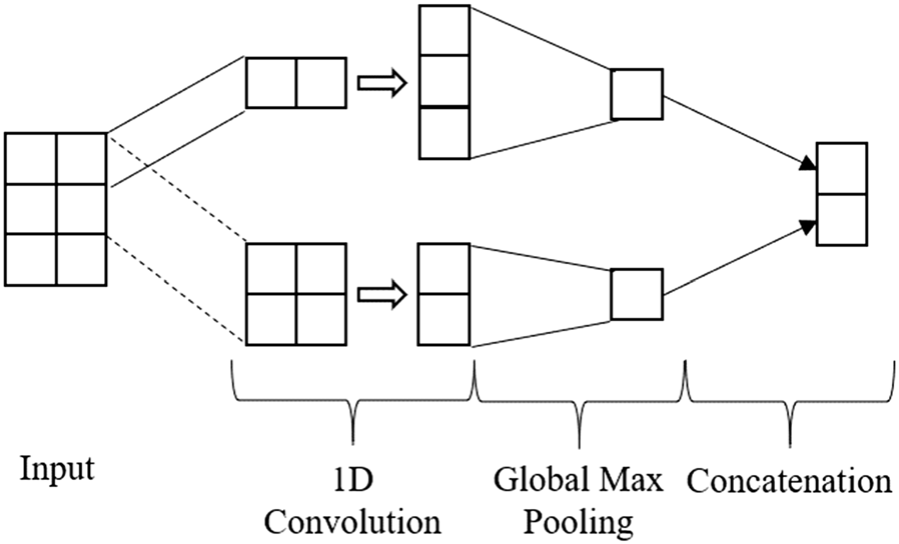
Diagram illustrating a single 1D CNN. The layer consists of 2 filters of size 1 and 2, respectively

*Single-Layer CNN:* contains a single layer with varying filter sizes that are then down-sampled by the global max-pooling layer and merged in a concatenation layer. The concatenation is then connected to a fully connected FFNN that outputs the probability of each class. Fig. 3 provides an example of a single one-dimensional convolution layer.

**Fig. 4:**
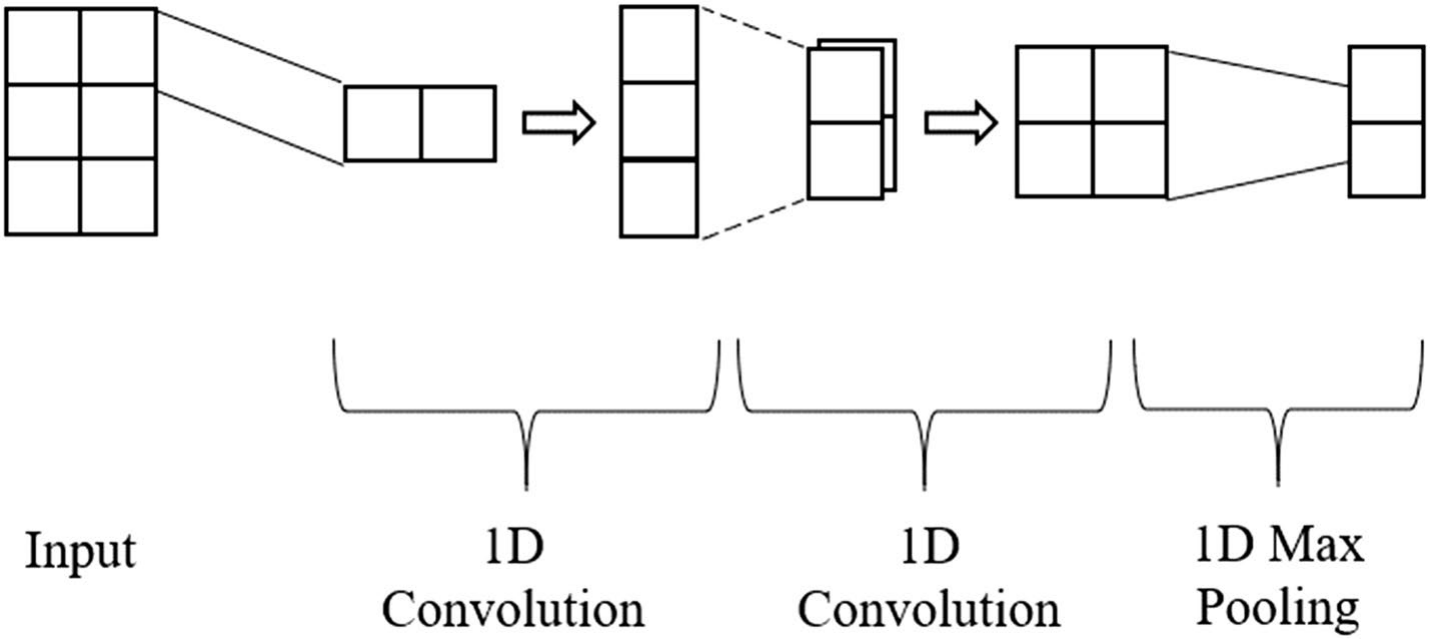
Diagram illustrating a 2-layer stack 1D convolution network. The first 1D convolution has 1 filter of size 1, generating a 3 - 1 feature map. A second convolution takes the 3 - 1 feature map and applies a 1D convolution with 2 filters of size 2, generating a 2 - 2 feature map. 1D max Pooling is then applied after the second convolution producing a vector

*Multi-Layer CNN:* contains multiple subsequent layers with varying filter sizes, followed by a one-dimensional max-pooling layer that is then connected to a fully connected classification network. Fig. 4 provides an example of the convolutional layers of a simple multilayer one-dimension CNN.

**Fig. 5:**
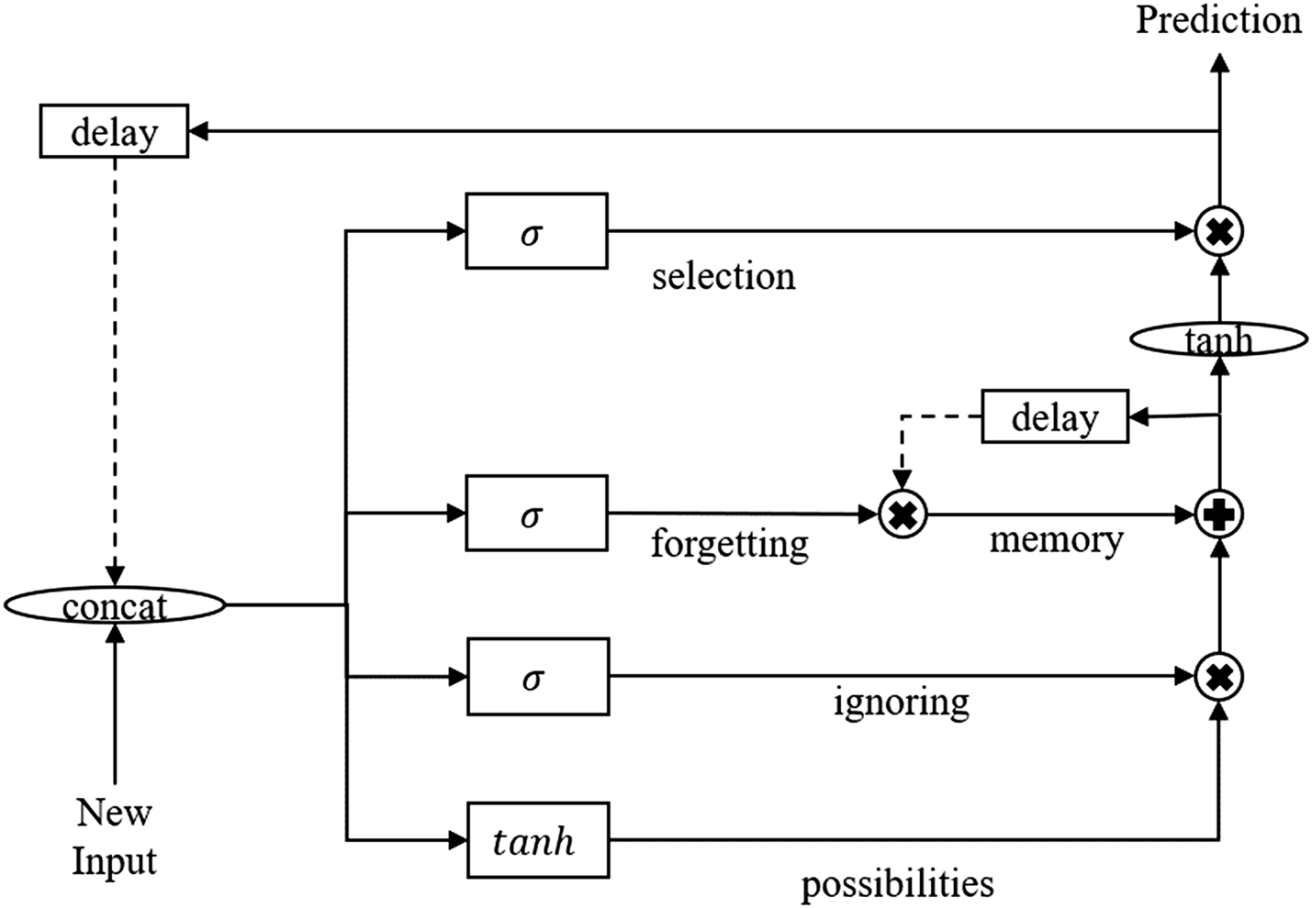
Diagram illustrating the LSTM architecture. The *tanh* and $\sigma$ gates are hidden layers with sigmoid activation function, + and *x* connections are pair-wise addition and multiplication operators, respectively. At the same time, the $\vert \vert$ connections are vector concatenation operators

**Fig. 6:**
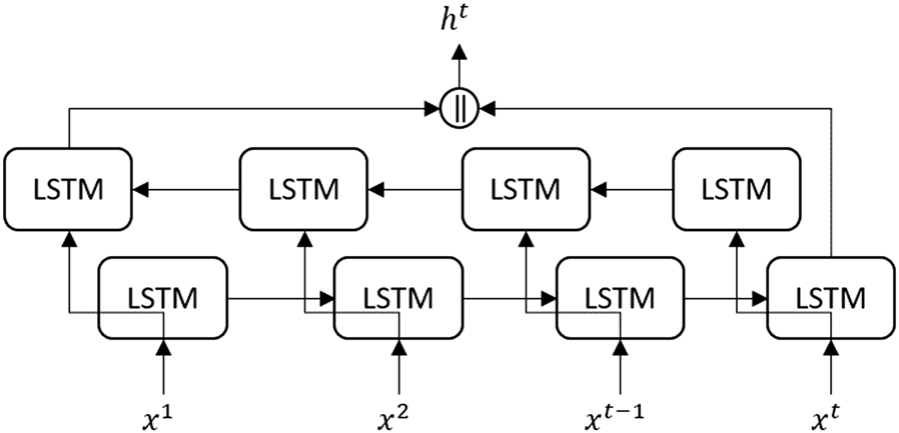
Diagram illustrating the Bidirectional LSTM architecture with the standard LSTM cell, as illustrated in Fig. 4

**Fig. 7:**
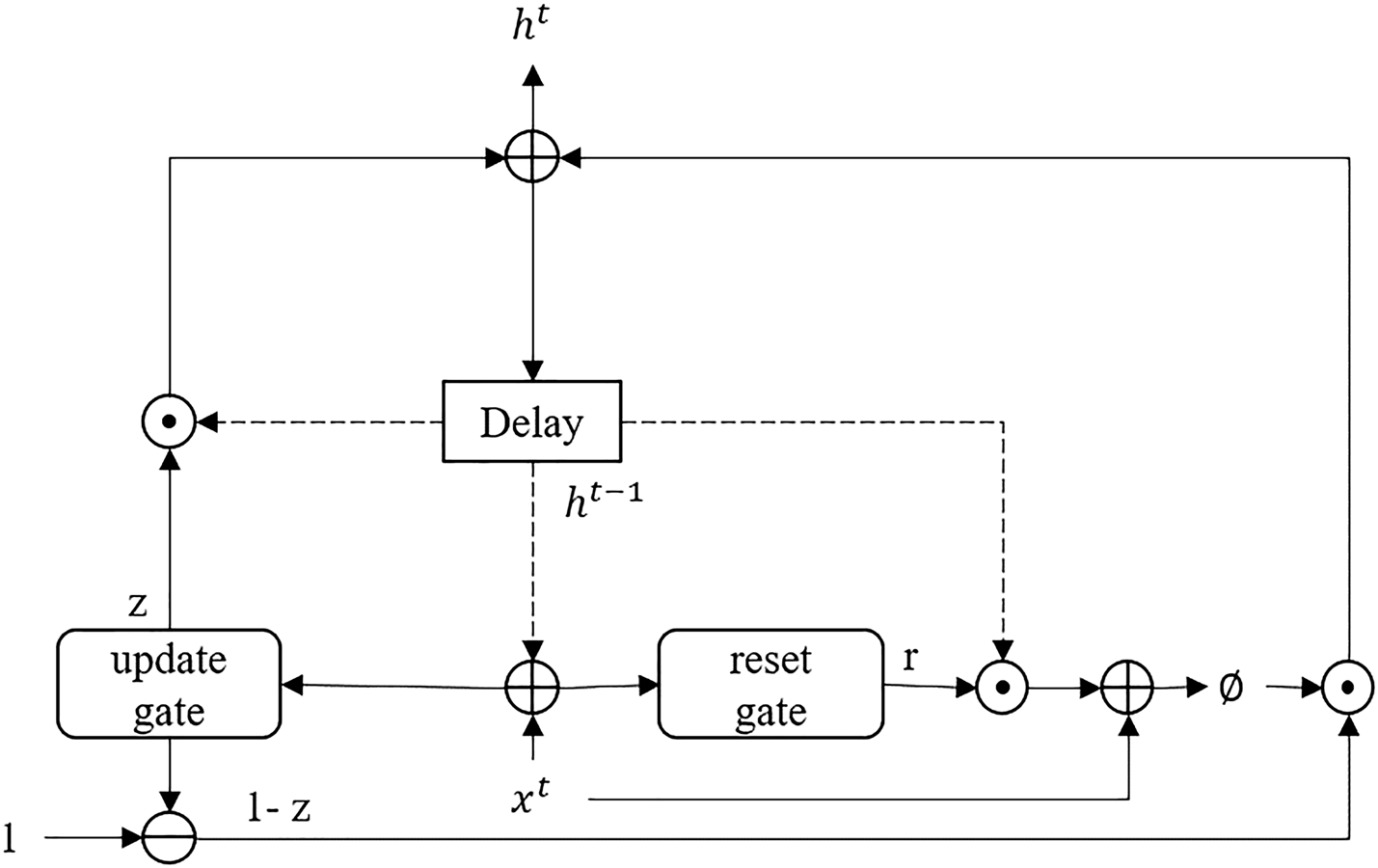
Diagram illustrating the GRU architecture. The reset and update gate are hidden layers with sigmoid activation function, + and *x* connections are pair-wise addition and multiplication operators, respectively. At the same time, the $\vert \vert$ connections are vector concatenation operators

*GRU, LSTM & bidirectional LSTM:* are recurrent neural network models that contain feedback loops to maintain various states of the memory. State memory is used to maintain important information from prior temporal predictions via gating mechanics (i.e., forget, ignore/retain, and prediction gates), as illustrated in Fig. 5. In a bidirectional LSTM, temporal input information is read in a sequential forward order and backward order, as illustrated in Fig. 6. For example, forward input would be read as $x^0$, $x^1$...$x^{n-1}$, $x^n$ and backward as $x^n$, $x^{n-1}$... $x^1$, $x^0$ where $x^0$ is the first input in the sequence and xn is the last input in the sequence. Unlike LSTM and Bidirectional LSTM, GRU contains a simplified architecture with only forgetting and updating gates. This allows GRU to consolidate the process of prediction and to maintain a memory cell. Instead, GRU utilizes a single internal state that is used to predict and maintain memory information via the update and reset gates, as illustrated in Fig. 7 [58–60].

*TCN:* While traditional CNN addresses spatial dependency, there is a limitation to temporal dependency within its architecture. As such, CNN has not seen the same dominance in perform in natural language processing tasks as it has in image processing. Temporal Convolutional Network (TCN) looks to address the temporal limitation in traditional CNN via an attention mechanism that allows convolution to focus on a specific region of interest. This is achieved via dilated casual convolutions such that the convolution achieves the following properties:

- Larger receptive field than the linear size depth of the network, allowing the network to retain a longer sequence of history
- Output at time t is convolved with only information from time t and earlier

**Fig. 8:**
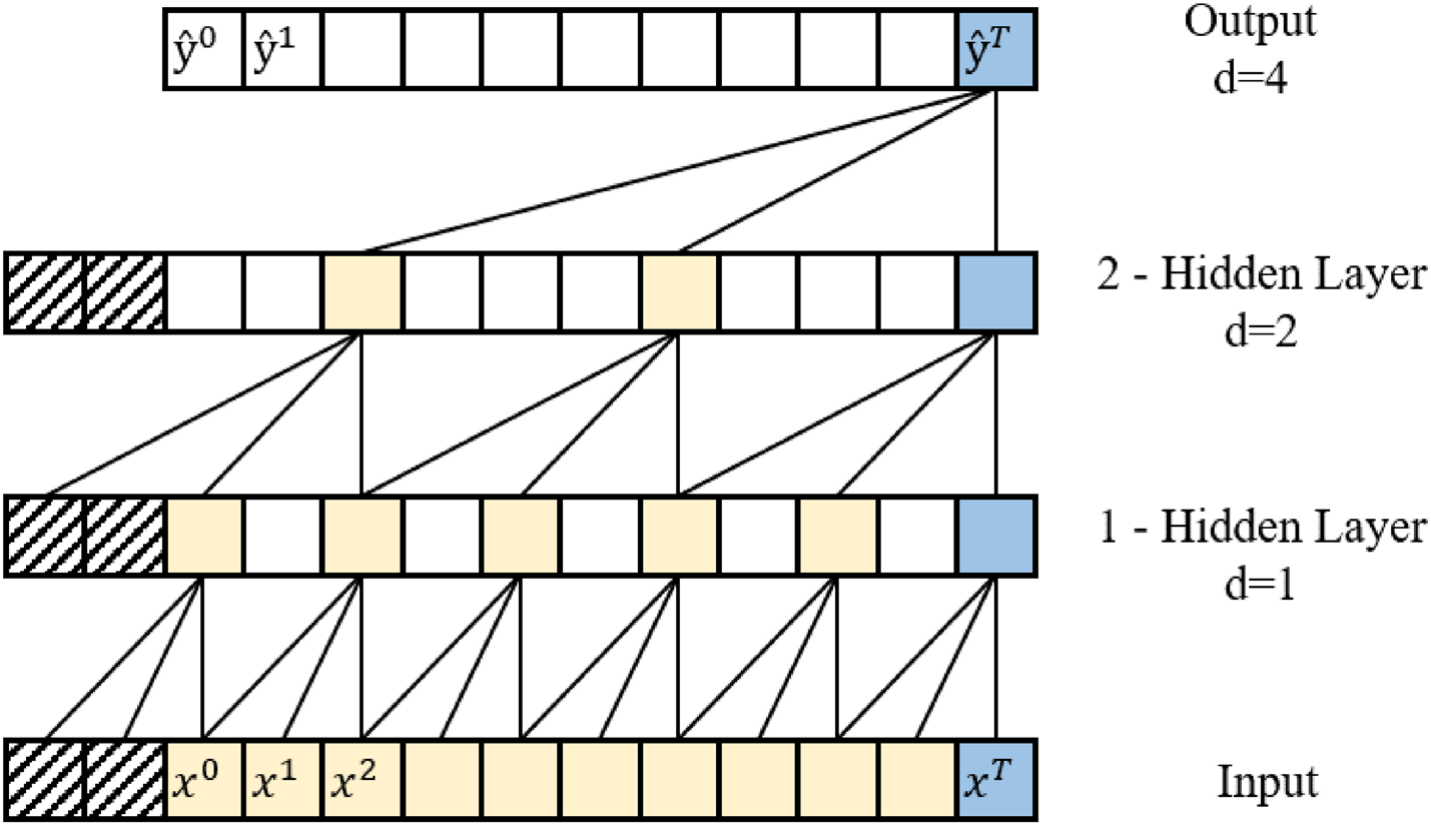
Diagram rendered from [58] illustrating dilated casual convolution layers from of filter size 3 and increase dilation of (1, 2, 4). At the second hidden layer, a neurons field of view is 6 (i.e., its scope allows it two see up to 6 input sequence within the past)

It can be seen in Fig. 8 that increasing d exponentially at subsequent layers allows the network to retrain longer memory dependency. As such, an increase in network size and/or filter size results in long-term dependency. Vice versa, short-term dependency can be achieved with smaller filter size and shallower networks [61].

### Ensemble

The last component of the automated sentiment analysis system is the ensemble system that combines the classification of the neural network models to generate a consolidated classification. Traditional ensemble approaches focus on applying a ’*hard*’ or ’*soft*’ voting system to consolidate the output of different models to produce a majority vote. In the case of ’*hard*’ voting, the concrete classification (i.e., ’*positive*’, ’*negative*’, or ’*neutral*’) is taken into consideration, whereas ’*soft*’ voting considered the probability distribution of each class (i.e., how confident the model perceives the tweet as being ’*positive*’, ’*negative*’, or ’*neutral*’). However, in either case, each model has an equal influence on the final decision. Intuitively, one can argue that in cases where experience and knowledge influence the correct decision, the weight of each vote should be treated differently. For example, in the case of a model, when voting class A is 80% correct, but voting class B is only 5% correct of the time. Therefore, in a situation such as this, the model has a high precision for class A. However, low precision with class B. Therefore, when considering the vote from the model, the model’s vote would be considered more when its vote is A, but its influence will be much lower when its vote is B. Based on the principle that the votes should be weighted depending on the situation, this body of work deviates from the standard ensemble approach.

Instead, the ensemble system extends hard and soft voting via an FFNN that learns the influential pattern that each model has towards the final decision. Hard voting values (one-hot encoding of the class prediction) or soft voting values (probability distribution of the class prediction) of each model is used as input to the FFNN. Since varying influence from the varying votes from the model can lead to the ideal decision, the complexity of the pattern could present a non-linear problem; therefore, a FFNN is implemented for the ensemble system.

## Implementation

### Dataset

Two different datasets are used in this work for training, testing, and evaluation. These sets include a Concussion Tweets-2018 dataset that we gathered and labeled in our lab, and SemEval-2016 datasets for pre-training. We also test our system against Concussions Tweets FIFA WC-2018 data set that we acquired during the FIFA world cup 2018.

#### Concussion Tweets-2018

The primary datasets were composed of extending the library of labeled data from the original works in [30]. Additional tweets were gathered using Twitter’s Search API for the month of May 2018 that were then manually filtered and labeled by a group of experts in our research lab. The search queries used to retrieve additional tweets contained a combination of scientific (i.e., TBI, concussion, etc.) and colloquial terms (i.e., *out cold, clocked out*, etc.). The terms were derived from a codebook that was initially developed in the works of [30]. Each tweet was manually labeled by at least two experts to ensure exclusion of bias that may be introduced by just a single labeler. Indecisive or split labeled results were additional labelers further labeling until a majority voting of 50% or more was achieved. Firstly, the tweets were labeled based on relevance such that tweets not discussing sports-related concussions were discarded. Afterwards, tweets were labeled based on their sentiment ranking between concussion and other sport’s related topics. For example, tweets that indicated an awareness of concussion and prioritized its significance over other topics occurring in that sport were labeled positive. In the end, the concussion twitter dataset was expanded to 15,800 tweets, with a distribution of 47% *positive*, 17% *negative*, and 36% *neutral*.

#### SemEval-2016

The dataset contains a set of labeled tweets classifying the general sentiment of the tweet into 3-levels (*’positive’, ’negative’, or ’neutral’*). The dataset consists of tweets that have been gathered between July and December 2015. Tweets were filtered such that the topics being discussed where only the top 200 most popular topics during that period were kept. The dataset is made available by the SemEval-2016 Task 4: Subtask A competition [62].

#### Concussions Tweets FIFA WC-2018

In mid Jun-July 2018, we gathered tweets from the audience of the FIFA World Cup 2018 to measure their level of concussion awareness. We used the same search queries used to retrieve information with the same combination of scientific and colloquial terms in addition to FIFA World Cup 2018 related keyword (i.e., fifa2018, worldcup2018). In the end, the Concussion Twitter FIFA WC 2018 dataset contains 82,842 tweets.

## Experiments

We conducted three main sequential experiments to train, tune, evaluate, and configure our proposed system. Our trained model is then applied to evaluate the public opinion towards sports-related concussion during the FIFA World Cup 2018 by analyzing twitter posts about athlete head injury during the event. Training and evaluation for all experiments were performed using the standard 80% and 20% split for training and testing datasets, respectively. Tenfold cross-validation was performed on the training datasets to fine-tune the hyperparameters. The batch size, epoch size, learning rate, loss function, and optimization algorithm were configured to 100, 40, 0.001, Weighted Categorical Cross-Entropy, and Adam, respectively, for all models. The dropout rate was 50% for FFNN and 20% for all other models (CNN, RNN, and TCN). We discuss each experiment in more detail below.

### Experiment 1

In the first experiment, we train and evaluate each of the 7 different proposed neural network architectures with a varying number of hidden layers and filter size configurations on a general sentiment analysis task using the SemEval-2016 dataset. This experiment is conducted to identify candidate models with the efficacy of classifying the sentiments of tweets. The list of configurations is illustrated in Table 3. The configuration has been selected to evaluate a breadth of models of increasing complexity. The efficacy of a down-sampling mapping approach also evaluated as displayed by the last two configurations for FFNN. GRU, LSTM and Bi-directional LSTM are all evaluated with a single 205 hidden layer network to maintain the same dimension as the word embedding. As per TCN, a filter size of 3 was selected to ensure the model spans the whole scope of the input space. The dilation at a given layer can be calculated as $d^i=\ 2^i$, where *i* is the given layer. The dilation for a filter size of 3 will result in 8, since the model will contain 4 layers. We can then calculate the effective history or the input scope of a layer to its preceding layer as ${eh}^i=(k-1)d^i$, where *k* is the filter size, *d* is the dilation, *i* is the layer, and eh is the effective history [61]. This results in effective history values of (2, 4, 8, 16) at each layer of our model. Finally, the field of view at a given layer is calculated as ${fv}^i=\ \sum _{j=0}^{i}{eh}^i$. This provides a value of 30 at the final layer. Since the initial weights of a neural network contribute to the final performance of the model, each model was trained and evaluated 30 times with a newly randomized initial weight, with the highest performing iteration being recorded. A large iteration size of 30, allows for a good sample of different initial weights to be tested. In addition, more extensive sampling of different initial weights larger than 30 did not yield significant improvement.

**Table 3:** Configuration setting for each architecture

Model	Layer/Filter size
FFNN	[400, 400] [400, 400, 400] [775, 225, 75, 25]
Single layer CNN	[1, 2] [1, 2, 3] [3, 4, 5] [1, 2, 3, 4, 5]
Multi-layer CNN	[1, 2]
GRU	[205]
LSTM	[205]
Bi-Dir LSTM	[205]
TCN	[3]

### Experiment 2

Models with higher performance from experiment 1 were then selected and trained on the sentiment classification of sport-related concussion using the Concussion Tweets-2018 dataset. Since a total of 12 models were evaluated in experiment 1, only the top 5 models were selected for experiment 2. Since transfer learning has been shown to produce state-of-the-art performance, we also evaluate the efficiency of training the models on the SemEval-2016 dataset and fine-tuning the model on the Concussion Tweets-2018 dataset [63]. Each tweet was padded and truncated based on the average length of tweets in the Concussion Tweets-2018 dataset, yielding a 24-by-205 (24 words, 205 vectors per word) dimensional input to each model. Similar to Experiment 1, each model was trained and evaluated 30 times with a newly randomized initial weight.

### Experiment 3

Candidate models for the ensemble model was then selected from experiment 2 based on a performance threshold. The threshold is calculated from the mean performance of all the models, yielding a threshold value of $61.3\pm 0.4$. This resulted in an ensemble with seven models (3 single-layer CNN, 2 multi-layer CNN, and 2 LSTM models). Since the input to our FFNN ensemble model are the votes from the 7 models, a shallow network with a 2-layer down-sampling mapping structure is implemented. The principle to the down-sampling mapping is for each layer to learn the non-linear projection of the subsequent layer to a down-sample size until finally projecting onto the 3-level classification. Specifically, our FFNN models consist of two layers with 21 and 7 neurons for the first and second layers, respectively.

## Evaluation and results

### Evaluation metrics

The evaluation is primarily based on the true-positive, false-positive, true-negative, and false-negative metrics. We apply Precision, Recall and F1, but mainly our measurement for the effectiveness of our approach is F1, the same as the general practice in the sentiment analysis community since 2016 [62]:

2 $$\begin{aligned} \begin{aligned} {\text {Precision}}=\sum _{s}{\left( \frac{n^s}{N}\right) \frac{TP^S}{TP^S+FN^S}}, \end{aligned} \end{aligned}$$

where *s* is the given sentiment of one of the three classes (positive, negative, and neutral). *N* is the total number of tweets, $n^s$ is the total number of tweets with a ground-truth label of *s*, *TP* is the true positive of the sentiment, and *FP* is the false positive of the sentiment.

3 $$\begin{aligned} \begin{aligned} {\text {Recall}}=\sum _{s}{\left( \frac{n^s}{N}\right) \frac{TP^S}{TP^S+FN^S}}, \end{aligned} \end{aligned}$$

where $TP^s$ is the true positive of the sentiment, and $FN^s$ is the false positive of the sentiment.

The F1-score is measured based on the Recall and precision, providing the harmonic average between the two and is calculated as:

4 $$\begin{aligned} \begin{aligned} F1=2\times \frac{{\text {Pr{e}cision}}\times {\text {R e{c}all}}}{{\text {Pr{e}cision}}+{\text {Re{c}all}}}. \end{aligned} \end{aligned}$$

Precision is the weighted precision score for the three sentiments (*positive*, *negative*, and *neutral*) from (2), and Recall is the weighted recall score for the three sentiments (*positive*, *negative*, and *neutral*) from (3).

### Results

The discussion here is presented in three different sections, presenting different analyses conducted on the individual neural network models with the same datasets. The first analysis presents the comparative results of the varying neural network models evaluated in this body of work with that of other state-of-the-art models used in the same dataset. It is important to note that as following the point of comparison between our system and state of the art reported system, we used the Accuracy metric since it was reported in those bodies of works. However, the primary metrics of performance in this body of work is F1-Score, which is much more accurate and sensitive to different types of errors.

**Table 4:** Accuracy results SemEval-2016 dataset. Results of the external systems from the SemEval-2016: task a competition are compared with the results of the neural network models presented in the current body of work. While a total of 34 systems contributed to the competition, only four systems are illustrated in descending order for comparison [52]

Model	Layer/Filter Size	Accuracy (%)
FFNN	[400, 400] [400, 400, 400] [775, 225, 75, 25]	59.5 59.0 58.8
Single layer CNN	[1, 2] [1, 2, 3] [3, 4, 5] [1, 2, 3, 4, 5]	**61.7** **61.1** 60.2 60.6
Multi-layer CNN	[1, 2]	**62.2**
GRU	[205]	60.6
LSTM	[205]	**61.2**
Bi-Dir LSTM	[205]	**62.2**
TCN	[3]	59.6

External System		Accuracy (%)
aueb.twitter.sentiment		62.9
sensei-lif		61.7
unimelb		61.6
senti-sys		60.9
Baseline		**34.2**

Lastly, among all the experiments, the optimization of all the models used the F1-Score as its primary metric. As reported in Table 4, our systems, even with accuracy metric, is among the state-of-the-art systems with SemEval-2016 dataset.

**Table 5:** F1-Score results of models pre-trained on SemEval-2016 and fine-tuned on the concussion dataset. The original performance of the not pre-trained model is also illustrated for comparison. In addition, the top-performing models are bolded

Model	Layer/Filter Sizes	F1 (%) Non-Pre-trained	F1 (%) Pre-trained SemEval-2016
Single layer CNN	[1, 2] [1, 2, 3]	**62.01** **61.35**	60.76 **61.39**
Multi-layer CNN	[1, 2]	**61.21**	**60.93**
LSTM	[255]	**61.38**	**61.17**
Bi-Dir LSTM	[255]	60.78	60.66

Bold values indicates the top-performing models

The second analysis presents the comparison of the varying neural network models among themselves and illustrates the subset of optimal neural network architectures for sentiment analysis of Concussion Tweets 2018. The models in this experiment were chosen from the best-performed model in Table 4. Also, the effect of pre-training the networks with SemEval-2016 dataset on the performance of the system with the Concussion Tweets 2018 dataset examined (see Table 5).

**Table 6:** Results of the ensemble system

F1-Score (%)	Precision (%)	Recall (%)	Accuracy (%)
**62.71**	62.71	62.72	62.72

Bold values indicates the top-performing models

**Fig. 9:**
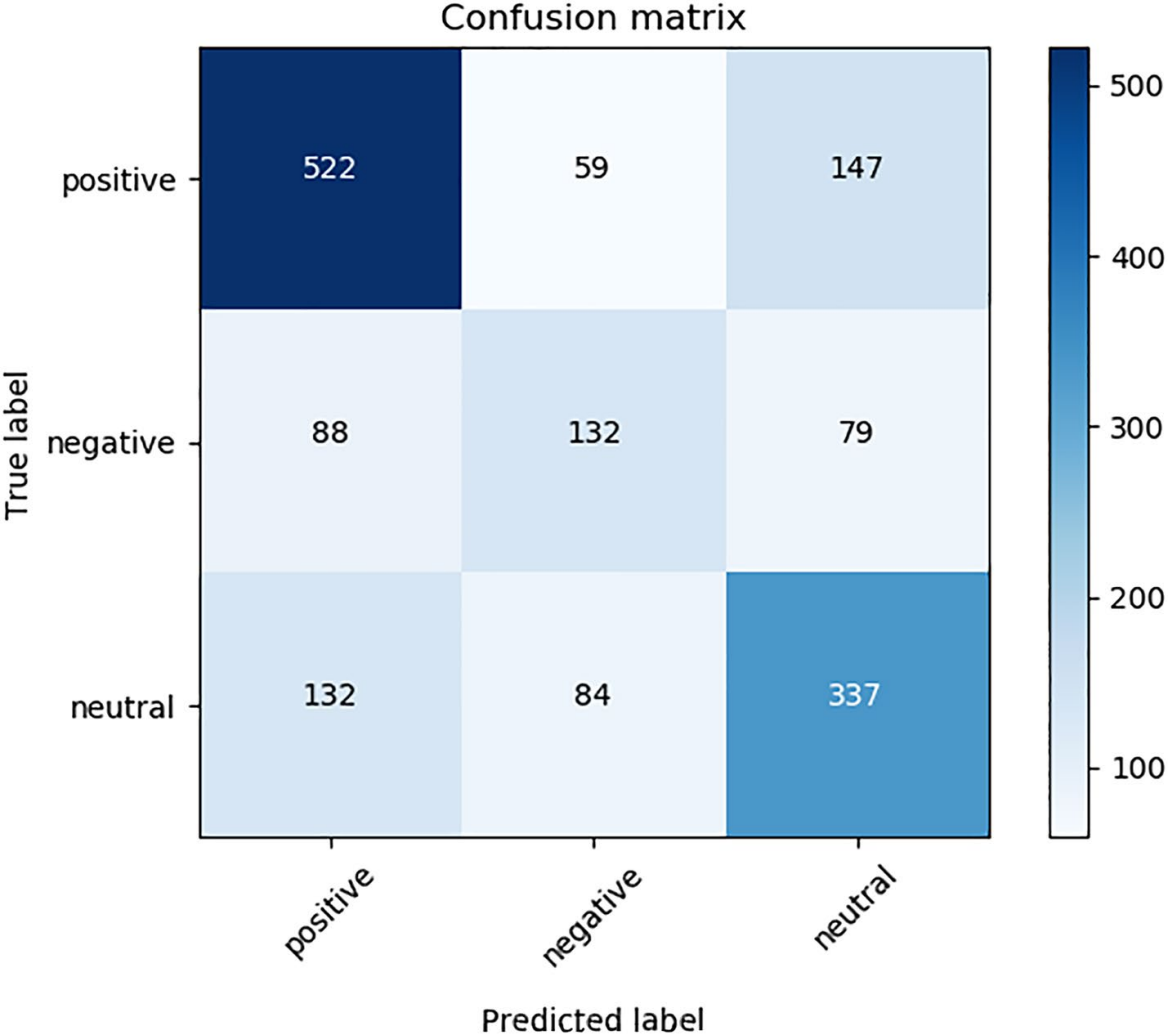
The confusion matrix of the ensemble model on the concussion dataset

In the third experiment, the performance of an ensemble model with all the best models (pre-trained and non-pre-trained) from the previous experiment was studied. A combination of 7 top-performing models (4 non-pre-trained models and 3 pre-trained models) was used to form an ensemble model with a Hard-voting approach, as illustrated in Table 5 via the bolded performance scores. At the end, ensemble model yielded the best result that we acquired, an F1-SCORE of 62.71%, as illustrated in Table 6. The confusion matrix of the ensemble model is also presented in Fig. 9. Also, we measure users’ tweets regarding the reaction to concussion in FIFA World Cup 2018. We processed 82,842 tweets regarding concussion in World Cup, and Table 7 presents our results. Some examples of the predictions from the FIFA World Cup 2018 datasets are presented in Table 8.

**Table 7:** Prediction Results of the FIFA World Cup 2018

Label	Total Number of Tweets	Percentage of Tweets
Positive	32,414	39.13%
Negative	21,563	26.03%
Neutral	28,865	34.84%

**Table 8:** Sample Predictions for FIFA World Cup 2018

Predicted label	Tweet
Positive	Is there a Concussion protocol in Soccer? #WorldCup #ENGBEL
Positive	Football should allow players to be temporarily substituted in case of concussion. Hope #Nigeria keeper ok. #WorldCup
Negative	Seen a lot of players faking head injury and theres another #worldcup
Negative	In football, if the ref gets hit by a player so hard that he’s knocked unconscious, what happens? #WorldCup #billasksfootballquestions
Neutral	You’d think he just lost 4 teeth and copped a concussion. #WorldCup
Neutral	It has just been confirmed that muslera had concussion #Worldcup #URUFRA

## Conclusion

In this work, user’s reactions to concussion in sports from the standpoint of tweets have been studied. It is important to note that all the tweets considered in this work have been related to both sport and concussion. The positivity, negativity, or neutrality of the tweets was examined with respect to the reaction to concussion in the tweet and not the sport itself. We gathered more than 15,000 tweets related to concussion in sports and labeled them by our medical experts in the mental health field. Rather than having just positive and negative in our approach, we asked our experts to label the data with three different tags, i.e, *Positive*, *Negative*, and *Neutral*. In addition, we measured our system based on F1-Score rather than precision or recall for a more accurate evaluation of our system. We applied seven different deep learning-based artificial neural networks with different parameters, equaling to twelve different systems, including four models of FFNN and three models of single-layer CNN, a model based on multi-layer CNN, model based on GRU, model based on LSTM, model Based on Bidirectional LSTM and a model based on TCN. We compared them against other states-of-the-art approaches using a public dataset, and among those, we chose the top five models and trained them with tweeter concussion 2018 dataset that are manually labeled and measure their performance. The effects of pre-training on those models were also examined. In addition, we used the ensemble model, which was the combination of the top seven models from the last stage, and used a hard voting algorithm to reach the best result (F1-Score of 62.71%) and outperformed the other models.

In the end, we measured the reactions of FIFA World Cup 2018 audience to a concussion. We gathered nearly 82,000 tweets during the FIFA World Cup 2018, and our system shows that an estimated 26% of them are negative tweets. This is a remarkably high number given the fact that years of work around concussions had been accomplished by the 2018 WC, including the institution of Zack Lystedt laws related to concussions in all 50 of the United States and other laws in other jurisdictions [21–23]. This suggests, an underlying public sentiment towards concussion that still requires more attention by future strategies aimed at reducing the frequency and burden of concussion. The presence of protocols such as those provided by the International Consensus Conferences on Concussion in Sport [17] need to be supported by more concerted multifaceted efforts including rule changes with important implications to players, coaches, teams and leagues that are strictly enforced. Our work demonstrates that scanning social media sites in the future with these sentiment analysis techniques could be a useful metric to gauge success of preventive measures, not only in concussion, but also in a number of areas of public health.

## Data Availability

The datasets used and/or analyzed during the current study are available from the corresponding author on reasonable request.
